# Serum sPD-L1 Levels in Early Pregnancy Predict Fetal Growth Restriction and Its Subtypes: A Prospective Nested Case–Control Study

**DOI:** 10.3390/ijms27115037

**Published:** 2026-06-02

**Authors:** Yao Wang, Yue Shi, Ruqun Zheng, Xiaoyi Bai, Maran Bo Wah Leung, Lai Kwan Lam, Chi Chiu Wang, Tak Yeung Leung

**Affiliations:** 1 Department of Obstetrics and Gynaecology, Faculty of Medicine, The Chinese University of Hong Kong, Hong Kong SAR, China; yaowang1@cuhk.edu.hk (Y.W.); shiyue@link.cuhk.edu.hk (Y.S.); ruqunzheng@link.cuhk.edu.hk (R.Z.); baixiaoyi@link.cuhk.edu.hk (X.B.); maranleung@cuhk.edu.hk (M.B.W.L.); laikwanlam@cuhk.edu.hk (L.K.L.); ccwang@cuhk.edu.hk (C.C.W.); 2 Li Ka Shing Institute of Health Sciences, The Chinese University of Hong Kong, Hong Kong SAR, China; 3 Hub of Obstetrics and Paediatric Excellence (HOPE), The Chinese University of Hong Kong, Hong Kong SAR, China; 4 The Chinese University of Hong Kong-Baylor College of Medicine Joint Centre for Medical Genetics, The Chinese University of Hong Kong, Shatin, Hong Kong SAR, China

**Keywords:** fetal growth restriction, blood-based biomarker, programmed death-ligand 1, placenta, disease prediction, pregnancy complications

## Abstract

Fetal growth restriction (FGR) is a leading cause of perinatal morbidity and mortality, yet reliable first-trimester biomarkers for early prediction remain lacking. Growing evidence suggests that placental dysfunction is a central pathological driver of FGR. Therefore, placenta-derived proteins in maternal circulation may serve as mechanistically informative biomarkers for early detection. Here, we aimed to evaluate several placenta-relevant molecules as biomarkers for predicting isolated FGR and its subtypes. In this prospective nested case–control study, we included singleton pregnancies that underwent Down screening in the first trimester and were subsequently diagnosed with FGR (n = 50, including early-onset FGR [EFGR] and late-onset FGR [LFGR]) and healthy pregnancies (n = 100). Pregnancies with maternal comorbidities or fetal anomalies were excluded. Maternal serum protein concentrations were measured using ELISA kits. There were no significant differences in placenta-specific protein 1 (PLAC1) or netrin-1 between the two groups. By contrast, maternal soluble programmed death-ligand 1 (sPD-L1) levels were significantly lower in overall FGR (*p* < 0.001) and FGR subtypes (*p* = 0.002) than in controls. Circulating sPD-L1 levels were positively correlated with gestational age at delivery and birth weight Z score. Each one-unit increase in sPD-L1 was associated with lower odds of overall FGR (Odd ratio, OR 0.33), EFGR (OR 0.17), LFGR (OR 0.43), birth weight Z score 3–10% (OR 0.30), and neonatal intensive care unit (NICU) admission (OR 0.38). Moreover, first-trimester sPD-L1 predicted overall FGR (area under the receiver operating characteristic curve, AUC 0.75), EFGR (AUC 0.84), LFGR (AUC 0.70), birth weight Z score 3–10% (AUC 0.75), and NICU admission (AUC 0.67). Collectively, decreased maternal circulating sPD-L1 in early pregnancy may serve as a potential biomarker for isolated FGR, warranting validation in larger multicenter mechanistic studies.

## 1. Introduction

Fetal growth restriction (FGR) is a common adverse birth outcome, with a global prevalence of 5% to 10% [1]. In low-income regions, the prevalence may reach 25% of pregnancies [2]. FGR is a leading contributor to perinatal morbidity and mortality [3]. Moreover, affected fetuses are at increased risk of long-term health complications, including neurodevelopmental impairment and adult-onset metabolic or cardiovascular disease [4,5]. However, robust early predictive biomarkers for FGR remain lacking, hindering timely identification of high-risk pregnancies and limiting opportunities for optimal monitoring and individualized management [6].

Although FGR is associated with multiple mechanisms, including maternal cardiovascular disease, genetic abnormalities, and environmental exposures [7,8,9], accumulating evidence suggests that placental dysfunction is a major contributor. Defective placentation, characterized by impaired trophoblast invasion and incomplete spiral artery remodeling, leads to vascular malperfusion, chronic hypoxia, oxidative stress, and a proinflammatory placental state, ultimately causing placental insufficiency and impaired fetal growth [10,11,12]. Moreover, histopathological studies consistently link impaired villous development to an increased incidence of FGR [13,14]. These findings raise the possibility that placenta-specific proteins involved in placental biology and function may provide mechanistic insight and serve as potential biomarkers for FGR.

Interestingly, several key molecules that regulate placental function have recently been identified and may be involved in FGR onset. Programmed death-ligand 1 (PD-L1) is abundantly expressed on cytotrophoblasts, syncytiotrophoblasts, and decidual cells [15], where it engages PD-1 on maternal immune cells to establish fetomaternal immune tolerance and protect the semi-allogeneic fetus from rejection [16]. Moreover, a recent study indicated that placental PD-L1 also promotes trophoblast migration and invasion [17]. Placenta-specific protein 1 (PLAC1) is a trophoblast-enriched membrane-associated protein expressed exclusively in the human placenta [18]. It plays a crucial role in placental development by promoting trophoblast proliferation, differentiation, and migration [19]. Clinical studies have associated abnormal PLAC1 expression or circulating PLAC1 mRNA levels with adverse pregnancy outcomes such as implantation failure, preeclampsia, and FGR [19,20]. Moreover, experimental data have shown that loss or dysregulation of PLAC1 impairs placental morphogenesis and function, leading to placentomegaly in dams and fetal growth restriction [21]. Additionally, netrin-1 is a newly identified protein expressed in trophoblasts and decidual stromal cells [22]. Beyond its classical role in neural development [23], netrin-1 promotes spiral artery remodeling and placental angiogenesis during pregnancy [24,25]. Aberrant netrin-1 expression has been associated with gestational diabetes mellitus (GDM) and FGR in humans [26,27,28]. Collectively, given their roles in immune regulation, trophoblast biology, and vascular remodeling, these three proteins may form a mechanistic axis linking placental dysfunction to FGR. Importantly, all are detectable in blood samples [29,30,31], highlighting their potential as circulating biomarkers of FGR.

Here, we conducted a prospective nested case–control study to determine whether sPD-L1, PLAC1, and netrin-1 are potential biomarkers associated with FGR. We enrolled women with isolated FGR and healthy pregnancies in the first trimester. The primary objective was to evaluate differences in serum levels of placenta-related proteins between the two groups. We further assessed their predictive performance and potential clinical utility for FGR screening. 

## 2. Results

### 2.1. Characteristics of the Participants

The clinical characteristics of patients with fetal growth restriction (FGR, n = 50) and healthy pregnancies (HP, n = 100) are shown in Table 1. Maternal age (*p* = 0.337), BMI, and gestational age at sample collection were comparable between the two groups. Compared with healthy pregnancies, patients with FGR had a twofold higher incidence of previous adverse pregnancy outcomes (*p* = 0.010), together with significantly lower gestational age at delivery, neonatal birth weight, and birth weight Z score (*p* < 0.001).

### 2.2. Maternal Circulating sPD-L1 Levels Are Reduced in Women with FGR Compared with Healthy Pregnant Controls in Early Gestation

**Figure 1:**
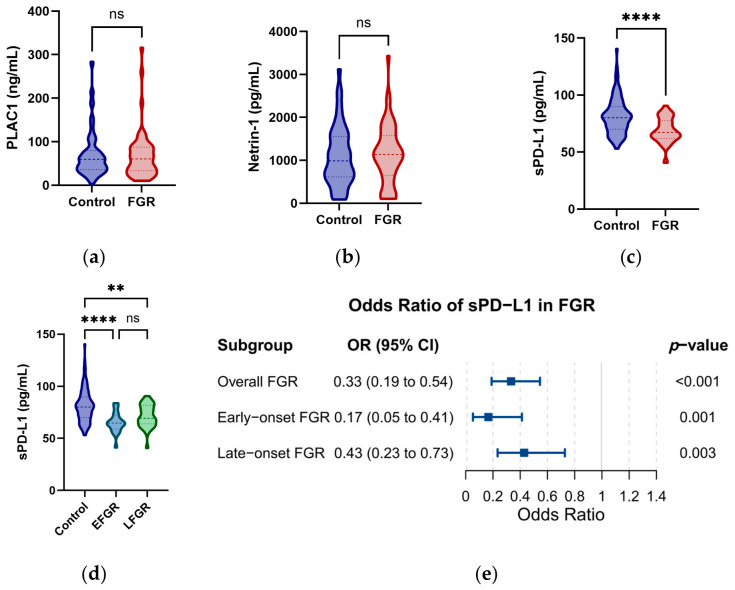
Novel biomarkers in FGR and FGR subtypes (**a**–**e**). Serum PLAC1 (**a**) and netrin-1 (**b**) were comparable between the FGR group and healthy pregnant controls, whereas serum sPD-L1 levels were significantly lower in women with (**c**) overall FGR and (**d**) FGR subtypes than in healthy pregnant controls. (**e**) ORs indicate an inverse association between sPD-L1 and FGR or its subtypes. ORs were obtained by logistic regression after adjustment for maternal age, body mass index (BMI), and gestational age. Abbreviations: placenta-specific protein 1 (PLAC1), soluble programmed death-ligand 1 (sPD-L1), fetal growth restriction (FGR), early-onset fetal growth restriction (EFGR), late-onset fetal growth restriction (LFGR), and odds ratios (ORs). ** *p*-value < 0.01, **** *p*-value < 0.0001, ns not significant.

Maternal circulating immunoregulatory and placental growth factors were first quantified using ELISA methods. There were no differences in PLAC1 or netrin-1 levels between the two groups (Figure 1a,b). By contrast, maternal sPD-L1 levels were 67% lower in FGR cases (26.96 ± 14.70) than in HP controls (39.69 ± 20.76; *p* < 0.001) (Figure 1c). After adjustment for confounding factors, including maternal age, gestational age, and BMI, the difference in sPD-L1 levels between groups remained significant, whereas no significant differences were observed for PLAC1 or netrin-1 (Supplementary Table S1). Notably, analyses stratified by FGR subtype confirmed that sPD-L1 levels remained significantly lower in both the EFGR and LFGR subgroups than in HP controls (*p* = 0.002) (Figure 1d). Consistently, the odds ratios (ORs) for serum sPD-L1 levels in overall FGR, EFGR, and LFGR were 0.33 (95% CI: 0.19 to 0.55, *p* < 0.001), 0.167 (95% CI: 0.053 to 0.413, *p* = 0.001), and 0.43 (95% CI: 0.23 to 0.73, *p* = 0.003), respectively, indicating that each one-unit (pg/mL) increase in serum sPD-L1 was associated with lower odds of overall FGR, EFGR, and LFGR (Figure 1e).

### 2.3. Maternal Serum sPD-L1 Levels Are Associated with Neonatal Birth Weight and NICU Admission Risk

**Figure 2:**
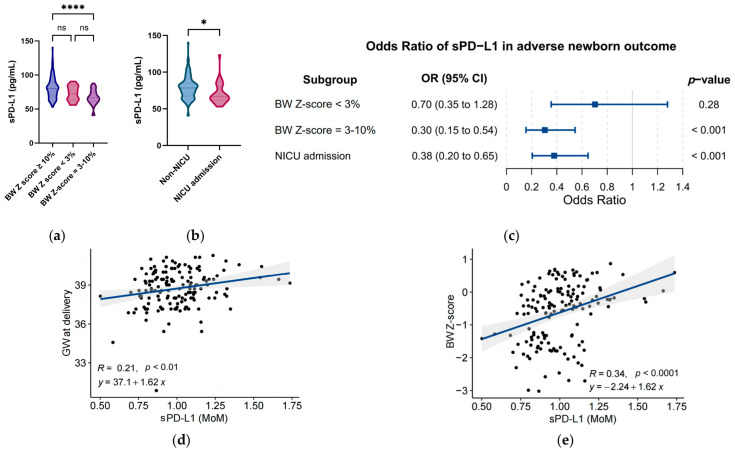
Serum sPD-L1 levels are significantly lower in women whose newborns had (**a**) a birth weight Z score of 3–10% and (**b**) NICU admission than in healthy pregnant controls (HP). (**c**) ORs indicate an inverse association between sPD-L1 and adverse newborn outcomes. ORs were obtained by logistic regression after adjustment for maternal age, body mass index (BMI), and gestational age. Scatter plots demonstrate positive correlations between MoM-transformed serum sPD-L1 levels and (**d**) gestational age at delivery and (**e**) birth weight Z score. Maternal age, body mass index (BMI), and gestational age were adjusted in the correlation analyses. Abbreviations: soluble programmed death-ligand 1 (sPD-L1), fetal growth restriction (FGR), early-onset fetal growth restriction (EFGR), late-onset fetal growth restriction (LFGR), neonatal intensive care unit (NICU), birth weight (BW), and odds ratios (ORs). * *p*-value < 0.05; **** *p*-value <0.0001, ns not significant.

The association between maternal serum sPD-L1 and neonatal birth weight was further evaluated using birth weight Z score. Maternal serum sPD-L1 levels were significantly lower in women whose newborns had a birth weight Z score between the 3rd and 10th percentiles than in those with normal birth weight (*p* < 0.001; Figure 2a). Significantly lower sPD-L1 levels were also observed in women whose newborns were admitted to NICU (*p* = 0.01; Figure 2b). In binary logistic regression analysis, sPD-L1 was inversely associated with birth weight Z score between 3% and 10% and with NICU admission, with ORs of 0.31 (95% CI: 0.16 to 0.55, *p* < 0.001) and 0.38 (95% CI: 0.20 to 0.65, *p* < 0.001), respectively, indicating that each one-unit (pg/mL) increase in serum sPD-L1 was associated with approximately 70% lower odds of birth weight Z score between 3% and 10% and around 60% lower odds of NICU admission (Figure 2c). Consistently, multiple of the median (MoM)-transformed sPD-L1 levels showed significant positive correlations with gestational age at delivery (r = 0.21, *p* = 0.010) and birth weight Z score (r = 0.34, *p* < 0.001) (Figure 2d,e). Taken together with the FGR analyses, these findings support decreased maternal sPD-L1 as a feature of impaired fetal growth.

### 2.4. Serum sPD-L1 Predicts FGR Onset and Adverse Neonatal Outcomes

**Figure 3:**
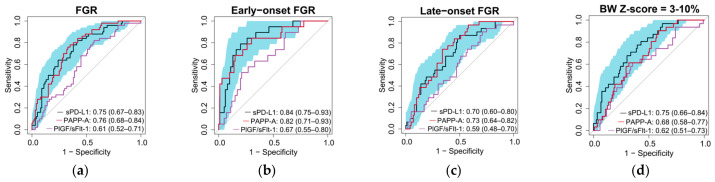
Serum sPD-L1, PAPP-A, and PlGF:sFlt-1 for FGR and newborn outcome prediction (**a**–**d**). ROC curves from logistic regression (LR) models using maternal serum sPD-L1, PAPP-A, and PlGF:sFlt-1 to predict (**a**) overall FGR, (**b**) early-onset FGR, (**c**) late-onset FGR, and (**d**) newborns with birth weight Z score of 3–10%. Shaded areas represent the 95% CI of the AUC estimates for sPD-L1-based prediction. Abbreviations: soluble programmed death-ligand 1 (sPD-L1), pregnancy-associated plasma protein A (PAPP-A), placental growth factor (PlGF), soluble fms-like tyrosine kinase-1 (sFlt-1), fetal growth restriction (FGR), early-onset fetal growth restriction (EFGR), late-onset fetal growth restriction (LFGR), and birth weight (BW).

To evaluate the potential of maternal serum sPD-L1 in early pregnancy for predicting FGR and adverse neonatal outcomes, logistic regression (LR) models based on serum sPD-L1 levels were developed. After adjustment for maternal age, BMI, and gestational age, the model showed good discrimination for FGR, with an area under the receiver operating characteristic curve (AUC) of 0.75 (95% CI: 0.67 to 0.83) for overall FGR, 0.84 (95% CI: 0.75 to 0.93) for EFGR, and 0.70 (95% CI: 0.60 to 0.80) for LFGR. We also measured serum pregnancy-associated plasma protein A (PAPP-A), placental growth factor (PlGF), and soluble fms-like tyrosine kinase-1 (sFlt-1) in the same cohort, as these are widely used biomarkers for FGR prediction [32,33]. Further comparisons showed that sPD-L1-based models outperformed the PlGF/sFlt-1 ratio for predicting overall FGR and its subtypes (AUC < 0.70 in all predictions, *p* < 0.05) and showed comparable performance to PAPP-A. Importantly, combining sPD-L1 with PAPP-A further improved prediction of overall FGR, achieving an AUC of 0.81 (Figure 3a–c, Table 2).

Moreover, for neonatal outcome prediction, serum sPD-L1 achieved an AUC of 0.75 (95% CI: 0.66 to 0.84) for identifying newborns with birth weight Z score between 3% and 10% (Figure 3d), whereas the AUCs were 0.64 (95% CI: 0.48 to 0.79) for predicting birth weight Z score < 3% and 0.67 (95% CI: 0.57 to 0.78) for predicting NICU admission (Supplementary Figure S1A,B). By contrast, the PlGF:sFlt-1 ratio yielded AUCs of 0.59 and 0.64 (*p* < 0.05), whereas PAPP-A achieved AUCs of 0.73 and 0.63 for the same outcomes, respectively, suggesting that sPD-L1 may have better performance for predicting severe low birth weight than PlGF:sFlt-1 and better performance for predicting NICU admission than PAPP-A. Taken together, these findings highlight the translational potential of serum sPD-L1 for predicting overall FGR, its subtypes, and neonatal complications.

### 2.5. sPD-L1 May Be Implicated in the Development of FGR Through Regulating PlGF and PAPP-A

**Figure 4:**
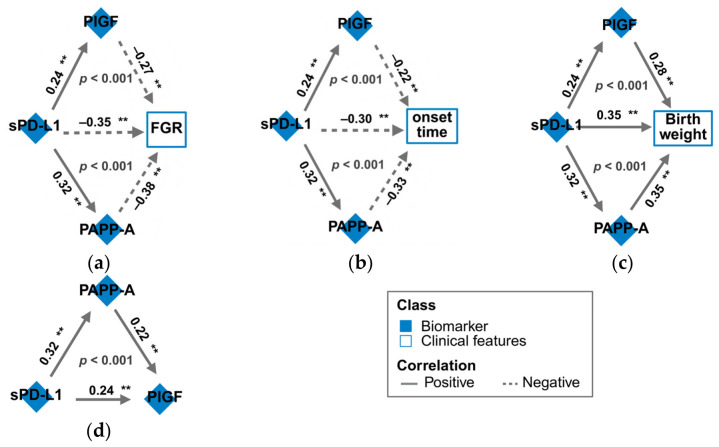
Mediation analysis of biomarkers and clinical features (**a**–**d**). The numbers along the edges indicate the partial correlation coefficients between variables, and the numbers in the center of each triangle indicate the significance of the mediation effect. Covariates (maternal age, body mass index [BMI], and gestational age) were adjusted in the analysis. ** *p*-value < 0.01. Abbreviations: soluble programmed death-ligand 1 (sPD-L1), pregnancy-associated plasma protein A (PAPP-A), placental growth factor (PlGF), and fetal growth restriction (FGR).

Mediation analysis was further performed to explore possible mechanisms by which sPD-L1 is associated with FGR-related neonatal outcomes (Figure 4). Consistent with the associations observed in the correlation and regression analyses, sPD-L1 was negatively correlated with FGR risk (r = −0.35, *p* < 0.001) and FGR onset time (r = −0.30, *p* = 0.006) (Figure 4a,b), whereas it was positively associated with birth weight (r = 0.35, *p* < 0.001) (Figure 4c). Moreover, PlGF and PAPP-A significantly mediated the associations of sPD-L1 with FGR status, onset time, and birth weight (*p* < 0.001 in all mediation analyses) (Figure 4a–c). In addition, the positive association between sPD-L1 and PlGF levels (r = 0.24, *p* < 0.001) was significantly mediated by PAPP-A (*p* < 0.001) (Figure 4d), suggesting potential regulatory relationships among these biomarkers. Taken together, these findings support a potential role of sPD-L1 in FGR-related adverse birth outcomes through mediation of the PlGF/PAPP-A axis.

## 3. Discussion

In this prospective cohort, women who later developed FGR had significantly lower sPD-L1 levels in early gestation than healthy pregnant controls, and this pattern persisted after stratification into early- and late-onset FGR. Consistently, lower first-trimester sPD-L1 levels were associated with increased odds of FGR, birth weight Z score 3–10%, and NICU admission and were correlated with earlier delivery and lower birth weight Z scores. Importantly, first-trimester serum sPD-L1 showed good predictive performance for overall FGR, outperforming PlGF:sFlt-1 in identifying pregnancies at risk and offering advantages for predicting neonatal complications. Collectively, these findings support sPD-L1 as a promising biomarker for future FGR risk stratification in prenatal care.

PD-L1 and its soluble form, sPD-L1, are increasingly recognized as central regulators of immune homeostasis beyond cancer [34]. During pregnancy, PD-L1 is highly expressed on trophoblast populations at the maternal–fetal interface [15]. Longitudinal studies have shown that maternal serum sPD-L1 increases throughout gestation, consistent with the progressive reinforcement of fetomaternal immune tolerance [35,36]. Mechanistically, trophoblast-derived sPD-L1 exerts broad immunoregulatory effects during pregnancy, including promotion of anti-inflammatory decidual macrophage phenotypes and trophoblast growth [17,37]. In vivo evidence suggests that PD-L1 blockade exacerbates embryo resorption and impairs fetomaternal tolerance in animal models [16]. In addition, sPD-L1 inhibits inflammatory cytokine production by peripheral blood mononuclear cells [35], thereby supporting the tolerant, low-inflammatory milieu required for normal placental development. Taken together, our finding of reduced sPD-L1 in women with FGR suggests that inadequate immune suppression in early pregnancy may contribute to FGR pathogenesis.

Aberrant sPD-L1 levels have been observed in various cancers and have been studied as prognostic biomarkers [38]. In pregnancy, our previous work showed that reduced placental PD-L1 expression and lower circulating sPD-L1 levels are associated with miscarriage [30,39]. Other studies of preeclampsia have likewise reported altered sPD-L1 concentrations compared with healthy pregnancies [40,41]. In addition, dysregulated placental PD-L1/PD-1 signaling has been associated with GDM and trophoblastic disease [15,42]. In the present study, we observed a 67% reduction in maternal sPD-L1 levels in women with FGR during the first trimester. Lower sPD-L1 levels were also associated with increased risk of low birth weight and earlier delivery. Notably, sPD-L1 showed superior discriminatory performance compared with established angiogenic markers, including sFlt-1 and PlGF, particularly for early-onset FGR (AUC = 0.84). Moreover, its predictive value was comparable to that of PAPP-A, which promotes trophoblast activity and fetal development [43]. Taken together, these findings suggest that sPD-L1 may serve as a broader indicator of maternal immune disequilibrium across pregnancy complications. Further large-scale longitudinal and mechanistic studies are needed to determine whether sPD-L1 could be incorporated into routine obstetric practice.

Interestingly, our mediation analysis suggested that sPD-L1 may participate in FGR development through the angiogenic factors PlGF and PAPP-A. This is consistent with experimental data showing that PD-L1 exerts intrinsic proangiogenic functions beyond its immune checkpoint role [44]. PD-L1 overexpression activates STAT signaling, enhances secretion of VEGF family ligands [45], and correlates with increased microvessel density. Similarly, VEGF activation is associated with PD-L1 expression [46], whereas PD-L1-directed VEGF/PlGF traps can normalize the stromal vascular compartment [47], suggesting that the PD-L1 axis is functionally embedded within canonical placental angiogenic pathways. In addition, similar to PD-L1 in pregnancy, PAPP-A is a key immunosuppressive factor involved in maintaining maternal–fetal tolerance [48]. Therefore, our findings raise the possibility that PD-L1 participates in an immune-angiogenic network in the placenta, thereby influencing placental vascular development. This interaction between PD-L1 and PlGF/PAPP-A signaling suggests a potential pathogenic pathway in FGR that warrants dedicated functional investigation.

A major strength of this study is its focus on first-trimester prediction of FGR, a setting in which robust biomarkers remain limited because of the need for long-term follow-up throughout gestation until delivery. Furthermore, a substantial proportion of FGR cases occur in the context of maternal hypertensive disorders, preeclampsia, and fetal genetic or structural abnormalities. By applying stringent exclusion criteria for maternal systemic diseases and fetal structural or genetic abnormalities, we focused on isolated FGR, thereby reducing confounding and better isolating the condition itself. Lastly, because we measured several placenta-related proteins with established roles in trophoblast function and placental homeostasis, our study provides not only predictive utility for FGR but also biologically plausible candidate targets for further mechanistic and translational investigation.

Our findings should be interpreted in light of several limitations. First, the biomarker panel evaluated in this study targeted placenta-related proteins and was assembled on the basis of existing literature and biological plausibility. Other immune and angiogenic mediators, such as TNF-α, IL-6, and VEGF, may also interact with these pathways in placental dysfunction. Future work adopting high-throughput omics-based approaches is needed for unbiased biomarker discovery and to more comprehensively characterize the complex molecular interactions underlying placental dysfunction and FGR. Second, although the sample size met the requirements of our power calculations, the data originated from a single tertiary referral center conducted in a Chinese population, and biomarker performance could vary across ethnic populations and clinical settings. Further validation in an independent cohort and replication in diverse populations is therefore needed. Moreover, regarding our mediation analysis based on clinical data, the proposed pathway requires validation in future mechanistic and experimental investigation. Lastly, as an observational study, our analysis remains susceptible to selection bias, which may affect the magnitude and generalizability of the observed associations.

In summary, despite the need for validation in larger prospective cohorts, our preliminary data suggest that serum sPD-L1 may have the potential to complement existing combined screening strategies for improving the early identification of pregnancies at risk of FGR and its subtypes.

## 4. Materials and Methods

### 4.1. Study Design and Human Participants

Our study was approved by the Joint Chinese University of Hong Kong-New Territories East Cluster Clinical Research Ethics Committee (CREC No. 2020.313). Pregnant women were recruited during routine first-trimester combined screening for trisomies (11^+1^ to 13^+2^ weeks of gestation), and written consent was obtained before enrollment. We identified 50 patients with confirmed FGR and 100 healthy pregnancies (HP) at the same gestational stage. The diagnostic criteria for FGR were based on the Society of Obstetricians and Gynaecologists of Canada (SOGC) Guideline [49]. Subjects with multiple pregnancies, fetal genetic or structural abnormalities, intrauterine death, miscarriage, antiphospholipid syndrome, diabetes mellitus, or hypertensive disorders of pregnancy were excluded. Additionally, pregnancies identified as high risk for preterm pre-eclampsia (risk > 1:100) by the FMF triple test, or those prescribed aspirin prophylaxis, were also excluded from the present analysis. Control pregnancies were matched for maternal ethnicity, age (within ±3 years), parity (nulliparous vs multiparous), maternal weight (within ±5 kg), calendar date of first-trimester screening (within ±30 days), and gestational age at blood sampling (within ±7 days), and had sex-adjusted birth weights between the 25th and 75th centiles according to the local reference population.

Basic clinical information was collected from the institutional electronic medical record system, including maternal variables (age, BMI, weight, parity, prior obstetric history, etc.). Gestational age was determined by ultrasound at 11 + 0 to 13 + 6 weeks of gestation. Pregnancy outcomes (gestational age at delivery, mode of delivery, birth weight, etc.) were followed until delivery. Birth weight Z scores were calculated using a locally developed gestational-age-specific reference. Percentiles for fetal abdominal circumference and estimated fetal weight were assigned using local population-based charts. Fasting blood samples were collected at the initial prenatal visit. Approximately 2 mL of peripheral venous blood was collected at this visit, centrifuged at 3000 rpm for 15 min, and stored in a −80 °C freezer until analysis.

### 4.2. Diagnosis of FGR

EFGR was defined as diagnosis before 32 weeks of gestation. At least one of the following three criteria had to be present: (1) an abdominal circumference or estimated fetal weight below the 3rd percentile; (2) late changes in umbilical artery Doppler assessment (i.e., absent or reversed end-diastolic velocity); or (3) a fetal abdominal circumference or estimated fetal weight below the 10th percentile accompanied by abnormal uterine artery Doppler findings (mean pulsatility index > 95th percentile) or abnormal umbilical artery Doppler findings (pulsatility index > 95th percentile) [49]. LFGR was defined as diagnosis at or after 32 weeks of gestation by either an abdominal circumference or estimated fetal weight below the 3rd percentile alone or at least two of the following three criteria: (1) an abdominal circumference or estimated fetal weight below the 10th percentile; (2) an abdominal circumference or estimated fetal weight crossing two quartiles; or (3) an abnormal Doppler finding, defined as an umbilical artery Doppler pulsatility index above the 95th percentile or a cerebroplacental ratio below the 5th percentile [49]. EFGR and LFGR were classified according to predefined clinical criteria and were considered prespecified subgroup categories in the study design.

### 4.3. Sample Size Calculation

The sample size was estimated based on the expected mean and standard deviation (SD) of serum PlGF concentrations in FGR and HP groups [50]. To achieve a power (1-β) of 0.8 at a significance level (α) of 0.05 with a 1:2 ratio, at least 37 patients with FGR and 74 HP women were required, as calculated using the Cleveland Clinic Sample Size Calculator for Clinical Study (https://riskcalc.org/samplesize/, accessed on 1 January 2020) [51].

### 4.4. Enzyme-Linked Immunosorbent Assay (ELISA) of Serum Levels of Proteins

Serum sPD-L1, PlGF, and sFlt-1 levels were measured using commercial R&D kits (Cat. DB7H10, Cat. DPG00, and Cat. DVR100C; R&D, Minneapolis, MN, USA). PLAC1 and netrin-1 levels in serum were assayed using ELISA kits from Cusabio Biotech (Cat. CSB-EL018108HU and Cat. CSB-E11899h; Cusabio Biotech, Wuhan, China). The sensitivity, limit of detection, and inter-assay coefficients of variation for these two kits are shown in Supplementary Table S2. Inter-plate assay variation (CV%) was controlled within 10%.

### 4.5. Correlation Analysis

ORs per unit increase in serum biomarker concentrations and 95% confidence intervals (CIs) were derived using a mixed-effects logistic model adjusted for maternal age, BMI, and gestational age with the *questionr* package (v.0.8.1) [52]. To investigate associations between biomarkers and gestational age at delivery or birth weight, ordinary least-squares linear regression based on Pearson correlation was performed using the *stats* package (v.4.5.1) with covariate adjustment [53]. Multiple of the median (MoM) values were used to standardize serum biomarker concentrations. Forest plots and fitted curves were visualized using *ggplot2* (v.4.0.0) [54].

### 4.6. Predictive Model Construction

We assessed the predictive value of the biomarkers using LR algorithm with the R packages *stats* (v.4.5.1) and *pROC* (v.1.19.0.1) [53,55]. Furthermore, an LR model was built from features that differed significantly between groups, and a stepwise strategy was used to exclude redundant variables. Predictive performance was evaluated by the AUC, sensitivity, and specificity. The optimal ROC cutoff value was estimated using Youden’s index. Differences between ROC curves for biomarkers were assessed using the DeLong test [56].

### 4.7. Mediation Analysis

Mediation analysis was conducted to further explore the relationships between independent variables (biomarkers) and dependent variables (clinical outcomes). The R package *mediation* (v.4.5.1) was used to estimate the average causal mediation effect, direct effect, total effect, and proportion mediated [57]. Bootstrapping was used to obtain robust CI for the indirect effects. A *p*-value < 0.05 for average causal mediation effect (ACME) was considered to indicate a significant mediation effect.

### 4.8. Statistical Analysis

Statistical analyses were performed and data were visualized using GraphPad Prism 10. Continuous and categorical variables were presented as mean ± SD and count (N, %), respectively. Demographic and clinical features of the case and control groups were tested for normality using the Kolmogorov–Smirnov test and for homogeneity of variance using Levene’s test. The potential effects of covariates, such as maternal age, BMI, and gestational age, on biomarker candidates were adjusted using analysis of covariance by fitting a general linear model. Comparisons of demographic and clinical features between the FGR and HP groups were performed with a two-tailed Student’s *t*-test using the R package *rstatix* (v.0.7.2) [58]. *p* < 0.05 was considered statistically significant.

### 4.9. Declaration of Generative AI and AI-Assisted Technologies in the Writing Process

ChatGPT 5.4 and Grammarly were used cautiously for linguistic and grammatical refinement to improve readability during proofreading. After using these tools, the authors reviewed and edited the content as needed and take full responsibility for the content of the publication.

## 5. Conclusions

In conclusion, our study identifies decreased maternal sPD-L1 in early pregnancy as a feature of impaired fetal growth. First-trimester sPD-L1 showed good discriminatory performance for overall FGR and outperformed established placental biomarkers such as PlGF and sFlt-1 in our cohort, indicating translational potential for the early prediction of adverse fetal growth outcomes. Furthermore, sPD-L1 may provide novel insight into angiogenic dysregulation in the pathophysiology of isolated FGR.

## Figures and Tables

**Table 1:** Demographic and clinical characteristics of the participants.

	Control		FGR		*p*-Value
	Mean	SD	Mean	SD	
n	100		50		NA
Maternal age (years)	31.83	4.22	31.14	3.91	0.34
BMI	20.78	3.06	20.84	3.18	0.91
Gestational age at sample collection (weeks)	12.37	0.46	12.22	0.37	0.07
Gravidity	2.06	1.13	2.04	1.34	0.92
Parity	0.48	0.67	0.48	0.65	>0.99
Previous adverse pregnancy outcome (%)	4 (4%)		8 (16%)		0.01
Gestational age at delivery (weeks)	39.25	1.03	37.680	1.65	<0.001
Birth weight (g)	3198.00	184.60	2311.00	380.00	<0.001
Birth weight Z-score	−0.06	0.42	−1.76	0.47	<0.001
Apgar score (1 min)	8.71	0.85	8.78	0.68	0.60
NICU admission (%)	13 (13%)		18 (36%)		0.001
PAPP-A (IU/L)	6.40	3.48	3.66	1.98	<0.001
hCG (IU/L)	65.52	47.47	61.61	44.05	0.63

Serum sPD-L1 levels were adjusted for maternal age, BMI, and gestational age. Abbreviations: fetal growth restriction (FGR), standard deviation (SD), body mass index (BMI), neonatal intensive care unit (NICU), and pregnancy-associated plasma protein A (PAPP-A).

**Table 2:** Predictive performance of biomarkers for FGR and adverse newborn outcomes.

Biomarker	AUC	95% CI	Sensitivity	Specificity	Threshold	*p*-Value
FGR						
sPD-L1	0.75	0.67–0.83	0.64	0.76	0.41	NA
PAPP-A	0.76	0.68–0.84	0.78	0.65	0.35	0.83
PlGF/sFlt-1	0.61	0.52–0.71	0.8	0.43	0.3	0.01
sPD-L1 + PAPP-A	0.81	0.74–0.88	0.62	0.86	0.49	0.06
Early-onset FGR						
sPD-L1	0.84	0.75–0.93	0.79	0.8	0.23	NA
PAPP-A	0.82	0.71–0.93	0.84	0.7	0.18	0.78
PlGF/sFlt-1	0.67	0.55–0.80	0.53	0.81	0.22	0.02
sPD-L1 + PAPP-A	0.88	0.79–0.96	0.79	0.85	0.21	0.41
Late-onset FGR						
sPD-L1	0.70	0.60–0.80	0.87	0.5	0.18	NA
PAPP-A	0.73	0.64–0.82	0.71	0.66	0.28	0.64
PlGF/sFlt-1	0.59	0.48–0.70	0.71	0.48	0.23	0.07
sPD-L1 + PAPP-A	0.77	0.68–0.85	0.87	0.56	0.19	0.10
BW Z-score < 3%						
sPD-L1	0.64	0.48–0.79	0.6	0.71	0.12	NA
PAPP-A	0.73	0.61–0.85	0.93	0.5	0.07	0.33
PlGF/sFlt-1	0.59	0.43–0.75	0.6	0.63	0.1	0.31
sPD-L1 + PAPP-A	0.74	0.63–0.85	0.87	0.60	0.09	0.24
BW Z-score = 3–10%						
sPD-L1	0.75	0.66–0.84	0.77	0.61	0.18	NA
PAPP-A	0.68	0.58–0.77	0.9	0.41	0.14	0.23
PlGF/sFlt-1	0.62	0.51–0.73	0.61	0.66	0.22	0.04
sPD-L1 + PAPP-A	0.77	0.69–0.85	0.84	0.61	0.17	0.27
NICU admission						
sPD-L1	0.67	0.57–0.78	0.71	0.66	0.22	NA
PAPP-A	0.63	0.52–0.74	0.58	0.69	0.23	0.41
PlGF/sFlt-1	0.64	0.54–0.75	0.74	0.57	0.2	0.57
sPD-L1 + PAPP-A	0.67	0.57–0.78	0.68	0.68	0.22	0.95

Prediction values were generated using logistic regression adjusted for maternal age, BMI, and gestational age. *p*-values were compared with sPD-L1. Abbreviations: fetal growth restriction (FGR), birth weight (BW), neonatal intensive care unit (NICU), soluble programmed death-ligand 1 (sPD-L1), pregnancy-associated plasma protein A (PAPP-A), placental growth factor (PlGF), soluble fms-like tyrosine kinase-1 (sFlt-1), area under the receiver operating characteristic curve (AUC), and confidence interval (CI).

## Data Availability

The de-identified clinical data underlying the main analyses are available in the Supplementary Material. Further data should be requested from the corresponding author, subject to institutional approval and applicable ethical requirements (tyleung@cuhk.edu.hk).

## Supplementary Materials

The following supporting information can be downloaded at: https://www.mdpi.com/article/10.3390/ijms27115037/s1.

## Abbreviations

The following abbreviations are used in this manuscript:

average causal mediation effect	ACME
area under receiver operating characteristic curve	AUC
birth weight	BW
body mass index	BMI
confidence interval	CI
early-onset fetal growth restriction	EFGR
enzyme-linked immunosorbent assay	ELISA
fetal growth restriction	FGR
healthy pregnancies	HP
late-onset fetal growth restriction	LFGR
logistic regression	LR
multiple of median	MoM
negative predictive value	NPV
odds ratios	OR
pregnancy-associated plasma protein A	PAPP-A
placental growth factor	PlGF
placenta-specific protein 1	PLAC1
positive prediction value	PPV
Society of Obstetricians and Gynaecologists of Canada	SOGC
small for gestational age	SGA
soluble fms-like tyrosine kinase-1	sFlt-1
soluble programmed death-ligand 1	sPD-L1
standard deviation	SD
