# Supplementary material for: Serum sPD-L1 Levels in Early Pregnancy Predict Fetal Growth Restriction and Its Subtypes: A Prospective Nested Case–Control Study
Source: Int J Mol Sci. 2026 Jun 2;27(11):5037. doi: 10.3390/ijms27115037 (PMC13257208; doi:10.3390/ijms27115037)
Supplement: Supplementary file 1 [file ijms-27-05037-s001.zip › ijms-4223026-supplementary-S1.pdf]

Table S1. Serum biomarker concentration between FGR and control groups

	**Control**		**FGR**		***P*-value**
	Mean	SD	Mean	SD	
n	100		50		NA
sPD-L1 (pg/mL)	81.39	15.67	68.99	11.05	<0.001
Adjusted sPD-L1 (pg/mL)	80.98	14.95	69.82	11.20	<0.001
PlGF (pg/mL)	39.69	20.76	26.96	14.70	<0.001
Adjusted PlGF (pg/mL)	39.02	19.55	28.29	13.72	0.001
sFlt-1 (pg/mL)	1316.00	541.90	944.30	427.30	<0.001
Adjusted sFlt-1 (pg/mL)	1320.51	527.68	934.39	419.66	<0.001
PlGF/sFlt-1	0.04	0.02	0.03	0.02	0.55
Adjusted PlGF/sFlt-1	0.03	0.02	0.03	0.02	0.96
PLAC1 (ng/mL)	70.41	54.06	69.05	57.35	0.89
Adjusted PLAC1 (ng/mL)	71.82	53.09	71.41	56.90	0.91
Netrin-1 (pg/mL)	1105.00	700.00	1148.00	692.40	0.73
Adjusted Netrin-1 (pg/mL)	1105.79	699.92	1148.96	691.72	0.73

Serum protein levels were adjusted for maternal age, BMI, and gestational age.

Abbreviation: fetal growth restriction (FGR), standard deviation (SD), body mass index (BMI), soluble programmed death-ligand 1(sPD-L1), placental growth factor (PlGF), soluble fms-like tyrosine kinase-1 (sFlt-1), placenta-specific protein 1 (PLAC1).

Table S2. Summary of ELISA kit performance

ELISA kit	Supplier Cat. No	Sensitivity	Limit of detection	Inter-assay coefficient of variation
Human sPD-L1	R&D, USA, DB7H10	25-1600 pg/mL	4.52 pg/mL	9.02%
Human PlGF	R&D, USA, DPG00	15.60-500.00 pg/mL	7.00 pg/mL	5.75 %
HumanVEGF R1/Flt-1	R&D, USA, DVR100C	31.30-2000.00 pg/mL	8.46 pg/mL	6.49 %
Human PLAC1	CUSABIO, CSB-EL018108HU	1.56 ng/mL-100 ng/mL	0.39 ng/mL	9.16%
Human Netrin-1	CUSABIO, CSB-E11899h	31.25 pg/mL-2000 pg/mL	7.81 pg/mL	8.21%

Abbreviation: soluble programmed death-ligand 1(sPD-L1), placental growth factor (PlGF), soluble fms-like tyrosine kinase-1 (sFlt-1), placenta-specific protein 1 (PLAC1).

Figure S1

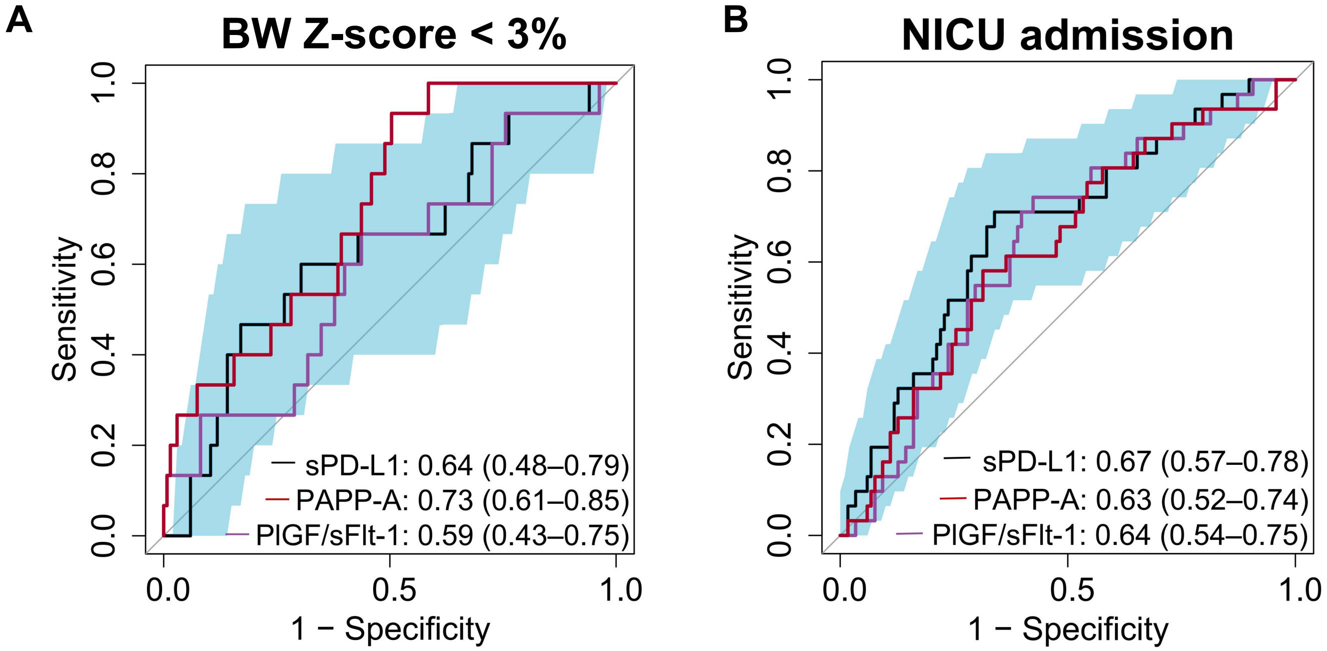

Figure S1. Serum sPD-L1, PAPP-A and PlGF: sFlt-1 for adverse newborn outcome prediction (A-B). ROC plots of logistic regression (LR) models using maternal serum sPD-L1, PAPP-A, and PlGF : sFlt-1 to predict newborn (**A**) birth weight Z score < 3% and (B) the risk of admission to NICU. Shaded areas represent the 95% CI of the AUC estimates of sPD-L1 prediction. Abbreviation: soluble programmed death-ligand 1 (sPD-L1), pregnancy-associated plasma protein A (PAPP-A), placental growth factor (PlGF), soluble fms-like tyrosine kinase-1 (sFlt-1), small for gestational weeks (SGA), neonatal intensive care unit (NICU).
