# Supplementary material for: Serum sPD-L1 Levels in Early Pregnancy Predict Fetal Growth Restriction and Its Subtypes: A Prospective Nested Case–Control Study
Source: Int J Mol Sci. 2026 Jun 2;27(11):5037. doi: 10.3390/ijms27115037 (PMC13257208; doi:10.3390/ijms27115037)
Supplement: Supplementary file 1 [file ijms-27-05037-s001.zip › ijms-4223026-supplementary-S2.pdf]

Supplemenatry File S1: The de-identified dataset underlying the main analyses

ID	Group	sPD-L1 (pg/mL)	ID	Group	sPD-L1 (pg/mL)	ID	Group	sPD-L1 (pg/mL)
1	Healthy pregnant control	69.5	51	Healthy pregnant control	81.78	101	Early-onset FGR	83.86
2	Healthy pregnant control	72.39	52	Healthy pregnant control	91.51	102	Early-onset FGR	72.31
3	Healthy pregnant control	102.6	53	Healthy pregnant control	73.06	103	Early-onset FGR	78.14
4	Healthy pregnant control	123.5	54	Healthy pregnant control	55.81	104	Early-onset FGR	58.77
5	Healthy pregnant control	86.74	55	Healthy pregnant control	80	105	Early-onset FGR	64.73
6	Healthy pregnant control	88.59	56	Healthy pregnant control	64.83	106	Early-onset FGR	73.06
7	Healthy pregnant control	97.98	57	Healthy pregnant control	71.1	107	Early-onset FGR	64.83
8	Healthy pregnant control	95.13	58	Healthy pregnant control	115.8	108	Early-onset FGR	64.73
9	Healthy pregnant control	58.77	59	Healthy pregnant control	63.03	109	Early-onset FGR	63.03
10	Healthy pregnant control	84.46	60	Healthy pregnant control	79.14	110	Early-onset FGR	65.49
11	Healthy pregnant control	73.35	61	Healthy pregnant control	104.3	111	Early-onset FGR	63.22
12	Healthy pregnant control	84.8	62	Healthy pregnant control	83.55	112	Early-onset FGR	82.67
13	Healthy pregnant control	81	63	Healthy pregnant control	69.31	113	Early-onset FGR	62.13
14	Healthy pregnant control	80.05	64	Healthy pregnant control	85.98	114	Early-onset FGR	56.72
15	Healthy pregnant control	106.4	65	Healthy pregnant control	140.4	115	Early-onset FGR	54.91
16	Healthy pregnant control	82.18	66	Healthy pregnant control	102.1	116	Early-onset FGR	55.81
17	Healthy pregnant control	82.91	67	Healthy pregnant control	82.94	117	Early-onset FGR	67.52
18	Healthy pregnant control	84.8	68	Healthy pregnant control	74.58	118	Early-onset FGR	64.83
19	Healthy pregnant control	62.69	69	Healthy pregnant control	68.52	119	Early-onset FGR	41.2
20	Healthy pregnant control	89.79	70	Healthy pregnant control	69.27	120	Late-onset FGR	75.27
21	Healthy pregnant control	82.94	71	Healthy pregnant control	80.66	121	Late-onset FGR	64.64
22	Healthy pregnant control	79.14	72	Healthy pregnant control	70.03	122	Late-onset FGR	68.52
23	Healthy pregnant control	73.35	73	Healthy pregnant control	79.14	123	Late-onset FGR	63.97
24	Healthy pregnant control	77.19	74	Healthy pregnant control	77.34	124	Late-onset FGR	87.64
25	Healthy pregnant control	117.3	75	Healthy pregnant control	83.55	125	Late-onset FGR	84.8
26	Healthy pregnant control	103.5	76	Healthy pregnant control	61.71	126	Late-onset FGR	61.71
27	Healthy pregnant control	65.62	77	Healthy pregnant control	61.23	127	Late-onset FGR	40.73
28	Healthy pregnant control	94.23	78	Healthy pregnant control	79.12	128	Late-onset FGR	84.8
29	Healthy pregnant control	107.3	79	Healthy pregnant control	72.88	129	Late-onset FGR	80.66
30	Healthy pregnant control	86.7	80	Healthy pregnant control	96.79	130	Late-onset FGR	82.18
31	Healthy pregnant control	66.59	81	Healthy pregnant control	74.67	131	Late-onset FGR	76.45
32	Healthy pregnant control	63.67	82	Healthy pregnant control	98.18	132	Late-onset FGR	85.22
33	Healthy pregnant control	82.18	83	Healthy pregnant control	88.86	133	Late-onset FGR	73.82
34	Healthy pregnant control	101.2	84	Healthy pregnant control	95.91	134	Late-onset FGR	90.55
35	Healthy pregnant control	110.1	85	Healthy pregnant control	83.55	135	Late-onset FGR	70.2
36	Healthy pregnant control	93.29	86	Healthy pregnant control	75.34	136	Late-onset FGR	81.78
37	Healthy pregnant control	78.14	87	Healthy pregnant control	71.55	137	Late-onset FGR	69.31
38	Healthy pregnant control	92.35	88	Healthy pregnant control	58.52	138	Late-onset FGR	66.62
39	Healthy pregnant control	80.66	89	Healthy pregnant control	63.93	139	Late-onset FGR	61.23
40	Healthy pregnant control	73.06	90	Healthy pregnant control	53.09	140	Late-onset FGR	68.52
41	Healthy pregnant control	79.1	91	Healthy pregnant control	89.74	141	Late-onset FGR	82.18
42	Healthy pregnant control	70.03	92	Healthy pregnant control	92.39	142	Late-onset FGR	67
43	Healthy pregnant control	79.9	93	Healthy pregnant control	77.34	143	Late-onset FGR	69.27
44	Healthy pregnant control	58.52	94	Healthy pregnant control	65.62	144	Late-onset FGR	60.19
45	Healthy pregnant control	72.88	95	Healthy pregnant control	82.67	145	Late-onset FGR	76.45
46	Healthy pregnant control	87.51	96	Healthy pregnant control	63.03	146	Late-onset FGR	61.23
47	Healthy pregnant control	83.7	97	Healthy pregnant control	90.63	147	Late-onset FGR	55.81
48	Healthy pregnant control	97.42	98	Healthy pregnant control	66.62	148	Late-onset FGR	59.43
49	Healthy pregnant control	62.13	99	Healthy pregnant control	76.45	149	Late-onset FGR	63.93
50	Healthy pregnant control	63.97	100	Healthy pregnant control	60.33	150	Late-onset FGR	77.34
